# Bacterial metabolites influence the autofluorescence of *Clostridioides difficile*

**DOI:** 10.3389/fmicb.2024.1459795

**Published:** 2024-10-08

**Authors:** Taylor D. Ticer, Anna M. Tingler, Janiece S. Glover, Sarah A. Dooley, Jacob Kendrick, Joseph P. Zackular, Suzanne Devkota, Gary D. Wu, Karley Mahalak, Amy Engevik, Melinda A. Engevik

**Affiliations:** ^1^Department of Microbiology & Immunology, Medical University of South Carolina, Charleston, SC, United States; ^2^Department of Regenerative Medicine & Cell Biology, Medical University of South Carolina, Charleston, SC, United States; ^3^Hollings Cancer Center, Medical University of South Carolina, Charleston, SC, United States; ^4^Department of Pathology and Laboratory Medicine, Perelman School of Medicine, University of Pennsylvania, Philadelphia, PA, United States; ^5^Department of Protective Immunity, Children’s Hospital of Philadelphia, Philadelphia, PA, United States; ^6^Department Division of Gastroenterology, Cedars Sinai, Los Angeles, CA, United States; ^7^Division of Gastroenterology and Hepatology, Perelman School of Medicine, University of Pennsylvania, Philadelphia, PA, United States; ^8^Dairy and Functional Foods Research Unit, United States Department of Agriculture, Washington, DC, United States

**Keywords:** *Clostridioides difficile*, *Klebsiella pneumoniae*, autofluorescence, metabolites, intestine

## Abstract

*Clostridioides difficile* is a bacterial pathogen that has been implicated in severe gastrointestinal infections. *C. difficile* has intrinsic green autofluorescence and the level of this autofluorescence is known to be increased by growth time and oxygen. Currently, it is unclear if dietary compounds or metabolites from the gut microbiota are able to enhance *C. difficile* autofluorescence. Here, we aimed to determine potential factors that affect *C. difficile* autofluorescence. After screening a large repertoire of compounds, we identified several substances, like L-lysine and pantothenate, that led to an increased *C. difficile* autofluorescence. We also found that several members of the gut microbiota, such as *Enterococcus faecalis, Klebsiella aerogenes* and *K. pneumoniae*, can increase *C. difficile* autofluorescence through their secreted compounds. We further focused on the effect of *K. pneumoniae* on *C. difficile* autofluorescence and found that multiple enteric strains of *K. pneumoniae* could enhance *C. difficile’s* autofluorescence. We used this enhanced autofluorescence to identify *C. difficile* in *K. pneumoniae* co-cultures by flow cytometry. Our findings shed light on the relationship between *C. difficile* and other members of the gut microbiota, as well as different factors that can affect *C. difficile* autofluorescence.

## Introduction

*Clostridioides difficile*, formerly known as *Clostridium difficile*, is an anaerobic, spore-forming bacterium responsible for a spectrum of gastrointestinal illnesses ranging from mild diarrhea to severe pseudomembranous colitis and toxic megacolon (Guh and Kutty, 2018; Guh et al., 2020). *C. difficile* infection is a significant cause of antibiotic-associated diarrhea, and it has become a major public health concern globally due to its increasing incidence, severity, and recurrence rates (Guh et al., 2017; Singh et al., 2017; Dong et al., 2023). Risk factors for *C. difficile* infection include recent antibiotic exposure, advanced age, immunocompromised status, prolonged hospitalization, and residence in long-term care facilities (Eze et al., 2017; Davies et al., 2020). The intricate interplay between host factors, environmental factors, and the gut microbiota all contribute to the severity and recurrence of *C. difficile* infection. There is an urgent need to understand the pathogenic traits of this organism to safeguard public health.

Autofluorescence can be useful for detecting and studying bacteria like *C. difficile* in laboratory settings. Several researchers have noted that *C. difficile* generates a green autofluorescence when excited with ultraviolet light (Ransom Eric et al., 2015; Buckley et al., 2016; Donnelly et al., 2022; Oliveira Paiva et al., 2022). This has made fluorescently tagging *C. difficile* with GFP difficult, but it is also advantageous because the autofluorescent capacity of *C. difficile* allows researchers to visualize the presence and distribution of the bacterium without the need for staining or other labeling techniques. It has been documented that growth stage and the presence of oxygen increases *C. difficile* autofluorescence (Oliveira Paiva et al., 2022), but other compounds that influence the autofluorescence of *C. difficile* have not been identified. Additionally, it is not clear if other gut bacteria influence the autofluorescence capacity of *C. difficile*.

In this study we sought to identify compounds that regulate *C. difficile* autofluorescence. We screened >300 compounds and identified several novel candidates that increased autofluorescence in *C. difficile*. We also screened several commensal and pathobiont members of the gut microbiota and found *Klebsiella* species in particular enhanced *C. difficile* autofluorescence. When *C. difficile* was grown with clinical isolates of *K. pneumoniae*, they had enhanced autofluorescence and could be separated by flow cytometry. These data provide new insights into *C. difficile* autofluorescence.

## Methods

### Bacteria and culture conditions

The following bacteria were selected for the experiments: *C. difficile* R20291, *Streptococcus mitis* NCIMB 13770, *Listeria monocytogenes* BAA 751, *Pseudomonas aeruginosa* ATCC 27853, *Enterococcus faecalis* ATCC 35038, *Morganella morganii* ATCC25830, *Klebsiella pneumoniae* ATCC 35657, *K. pneumoniae* CB1, *K. aerogenes* ATCC 35029, *Escherichia coli* Nissle 1917, *E. coli* K12, *Lactobacillus acidophilus* ATCC 4356, *Bifidobacterium breve* ATCC 15698, and *B. bifidum* ATCC 29521. *K. pneumoniae* strains C015, A14 CFH, and A05E were isolated from the creeping mesenteric adipose of Crohn’s disease patients, identified with full-length 16S rRNA sequencing, and kindly provided by Suzanne Devkota. *K. pneumoniae* strains Dynamic 35, UNK34, and Dynamic 18 were isolated from the stool of patients with *C. difficile* infection, identified with MALDI, and kindly provided by Karley Mahalak.

*C. difficile* was streaked plated onto cycloserine-cefoxitin fructose agar (CCFA) and single colonies were inoculated into brain-heart infusion (BHI) broth supplemented with 2% yeast extract and 0.2% cysteine (BHIS; FisherScientific). Cultures were grown at 37°C anaerobically in an Anaerobe Systems AS-150 anaerobic chamber overnight before use. *L. acidophilus* was streak plated onto De Man, Rogosa, and Sharpe (MRS; FisherScientific) agar plates and single colonies were inoculated into MRS broth and grown at 37°C anaerobically overnight before use. *B. breve* and *B. bifidum* were streak plated onto *Bifidobacterium* selective agar (Anaerobe Systems, Cat # AS-6423) and single colonies were inoculated into MRS broth and grown at 37°C anaerobically overnight before use. *S. mitis*, *L. monocytogenes*, *P. aeruginosa*, *E. faecalis*, *M. morganii*, *K. pneumoniae*, *K. aerogenes*, and *E. coli* were streak plated onto BHI agar and single colonies were inoculated into BHI broth and grown aerobically, shaking at 37°C overnight before use.

Overnight cultures of bacteria were grown in their above indicated conditions before being subcultured at an OD_600nm_ = 0.1 into 5 mL of a chemically defined media, ZMB1, supplemented with 100 mM glucose (Horvath et al., 2023). After 6 h of growth, the optical density of the bacterial cultures was examined at OD_600nm_ on a ThermoFisher Nanodrop using the cuvette function as a proxy of growth (Table 1). Cultures were centrifuged at 4,450 x g for 10 min to pellet bacteria. The cell-free supernatant was filtered with a 0.22 μm syringe filter to collect sterile, log-phase conditioned media. This supernatant was used immediately for autofluorescence assays.

**Table 1:** Optical density (OD600nm) of bacteria grown overnight in ZMB1.

Bacteria	OD600nm
*Stretococcus mitis* NCIMB 13770	1.1
*Listeria monocytogenes* BAA 751	1.9
*Pseudomonas aeruginosa* ATCC 27853	0.8
*Enterococcus faecalis* ATCC 35038	3.3
*Morganella morganii* ATCC 25830	1.5
*Escherichia coli* Nissle 1917	2.8
*Klebsiella pneumoniae* ATCC 35657	3.5
Klebsiella aerogenes ATCC 35029	4.8
*Escherichia coli* K12	2.7
*Bifidobacterium bifidum* ATCC 29521	0.3
*Bifidobacterium breve* ATCC 29521	0.5
*Lactobacillus acidophilus* ATCC 4356	0.3

### Autofluorescence measurements

To assess *C. difficile* autofluorescence over time, an overnight culture of *C. difficile* was subcultured into BHI at OD_600mm_ = 0.1 and was grown at 37°C anaerobically. At 1, 3, 6, 9, 12, 18 and 24 h post-inoculation, 1 mL samples were removed and centrifuged at 5,000 x g for 5 min to pellet the bacteria. The bacterial pellet was washed twice with sterile phosphate-buffered saline (PBS) and 100 μL of the washed *C. difficile* in PBS was transferred to a 96-well plate and the fluorescence in the green channel (excitation 485 nm/emission 528 nm) was read on a Biotek Synergy H1 plate reader. Blank PBS without *C. difficile* was included as a negative control. Biolog© Phenotypic Microarray plates PM1, PM2a, PM9, and PM10 were used to measure *C. difficile* autofluorescence with various compounds. Overnight cultures of *C. difficile* were subcultured into BHI at OD_600mm_ = 0.1 and grown at 37°C anaerobically for 6 h. *C. difficile* was then washed twice with PBS before being resuspended in PBS. To obtain baseline levels of autofluorescence in the Biolog microarray plates, we added 90 μL of PBS to the plate and read fluorescence in the green channel (485 nm/528 nm) on a Synergy H1 plate reader. We then added 10 μL *C. difficile* at OD_600nm_ = 10 to the Biolog microarray plates containing 90 μL of PBS; resulting in a final concentration of OD_600nm_ of 1.0 in PBS. The plates were incubated for five minutes before reading the green fluorescence (485 nm/528 nm) on a Synergy H1 plate reader. The reported fluorescence represents the values with the baseline fluorescence removed. To confirm the findings of the Biolog microarrays, we added concentrations of L-lysine (FisherSci) and pantothenate (FisherSci) ranging from 1–100 nM to 96-well plates and added *C. difficile* at an OD_600nm_ of 1.0. Similar to the Biolog plate assay, autofluorescence was measured after 5 min of incubation at excitation 485 nm/528 nm on a Synergy H1 plate reader.

To examine *C. difficile* autofluorescence in response to bacterial metabolites, we grew *S. mitis* NCIMB 13770, *L. monocytogenes* BAA 751, *P. aeruginosa* ATCC 27853, *E. faecalis* ATCC 35038, *M. morganii* ATCC25830, *K. pneumoniae* ATCC 35657, *K. aerogenes* ATCC 35029, *E. coli* Nissle 1917, *E. coli* K12, *L. acidophilus* ATCC 4356, *B. breve* ATCC 15698, and *B. bifidum* ATCC 29521 in 5 mL cultures of ZMB1 for 6 h and collected cell-free supernatant (see *Bacteria and Culture Conditions*). 100 μL of this cell-free supernatant was added to a 96-well DeepWell microplate (FisherSci #12-566-120) in quadruplicate and then 900 μL of *C. difficile* was added in ZMB1 at an OD_600nm_ = 0.1. The plate was grown anaerobically for 9 h and then 100 μL of culture was transferred via a multi-channel pipette to a 96-well conical bottom microplate (FisherSci # 277143). The plate was centrifuged at 2,000 x g for 10 min to pellet the bacteria. The plate was washed twice with PBS and the *C. difficile* bacteria were resupended in PBS and transferred to a 96-well flat bottom microplate (FisherSci #12-565-501). This plate was then read on a Synergy H1 plate reader at 485 nm/528 nm. The pH of the *C. difficile*-supernatant cultures was examined using a liquid litmus assay, as previously described (Engevik et al., 2023). Briefly, 200 μL of the *C. difficile* culture was transferred from the 96-well DeepWell microplate to a 96-well conical bottom microplate and the plate was centrifuged at 2,000 x g for 10 min to pellet the bacteria. Then 150 μL of the cell-free supernatant was transferred to a 96-well filter plate containing a 0.2 μm PVDF filter plate (FisherSci # MSIPS4510) and the supernatant was sterile filtered. 90 μL of the sterile supernatant was transferred to a 96-well flat bottom microplate and 10 μL of litmus (10 mg/mL) was added to the solution. The plate was read at 520 nm and 590 nm and the ratio was used to calculate the pH via a standard curve.

As confirmation that bacterial products could influence *C. difficile* autofluorescence, *E. faecalis* ATCC 35038, *K. pneumoniae* ATCC 35657, and *K. aerogenes* ATCC 35029 were grown in ZMB1 for 6 h and 25 μL of the cell-free supernatant from these cultures were added to 96-well flat bottomed plate. As a negative control, uninoculated ZMB1 was added to the plate as well. We then added 65 μL of PBS and read the baseline levels of autofluorescence on a Synergy H1 plate reader. After getting baseline values, we added 10 μL of a 6 h culture of *C. difficile* in PBS to the plate, incubated for 5 min and then read fluorescence on a Synergy H1 plate reader. This assay was repeated just with *K. pneumoniae* ATCC 25657 supernatant at various concentrations (3.125–100% supernatant).

### *Clostridioides difficile* and *Klebsiella pneumoniae* co-cultures

*C. difficile* and *K. pneumoniae* strains (ATCC 35657, CB1, C015, A14 CFH, A05E, Dynamic 35, UNK34, and Dynamic 18) were grown overnight as mono-cultures in BHI. The bacteria were then subcultured at an OD_600nm_ = 0.1 in 5 mL of ZMB1 supplemented with 100 mM glucose for mono-cultures or an OD_600nm_ = 0.05 in 5 mL of ZMB1 supplemented with glucose for co-cultures. Cultures were incubated anaerobically at 37°C for 6 h. After the incubation, cultures were centrifuged at 4,425 x g for 5 min to pellet the bacteria and the pellets were washed twice with PBS. The culture pellets were resuspended in PBS and read for green fluorescence (485 nm/528 nm) and OD_600nm_ using a Synergy H1 plate reader.

### Flow cytometry

*C. difficile* and *K. pneumoniae* were grown in mono- and co-cultures in ZMB1 anaerobically at 37°C for 20 h and then used for flow cytometry analysis. Bacteria were pelleted and resuspended in 4% paraformaldehyde (Alfa Aesar, Cat# J61899) and incubated at room temperature for one hour. Bacterial cultures were washed twice with PBS before being resuspended in 10 μg/mL of Hoechst. After 10 min of room-temperature incubation with Hoechst, the bacteria were washed with 0.2% BSA in PBS before being resuspended in 0.2% BSA in PBS. Bacteria were then run on a Beckman Coulter CytoFLEX LX flow cytometer. As bacteria are too small for the machine to detect unstained, the primary threshold was set to UV525 to detect the Hoechst stained bacteria. Autofluorescence was measured as B525-FITC. Analysis was performed using FlowJo version 10.

### gDNA isolation and qPCR

To confirm the presence of both bacteria in co-culture conditions, *C. difficile* and *K. pneumoniae* strains were grown in mono- and co-cultures in ZMB1 anaerobically at 37°C for 20 h. After 20 h, 1 mL of the culture was transferred to a microfuge tube and centrifuged at 5,000 x g for 5 min to pellet the bacteria. We isolated gDNA from the bacterial pellet using the Zymo Quick-DNA Fecal/Soil Microbe Kits according to the manufacturer’s instructions. Quantitative real time PCR (qPCR) was performed using a Bio-Rad CFX96 Real Time qPCR machine (Bio-Rad). Forward and reverse primers for *C. difficile* and *Klebsiella* were added to SYBR Green mastermix (Genesee Scientific #17-501DP) and gDNA. Bacterial colony forming units (CFUs) were calculated from CT values based on standard curves of *C. difficile* and *K. pneumoniae*.

### Fluorescent imaging

Images of *C. difficile* autofluorescence were obtained from cultures of *C. dififcile* in PBS incubated with 50% cell-free supernatant from *K. pneumonia* ATCC 35657 in ZMBI and co-cultures of *C. difficile* and *K. pneumoniae* ATCC 35657 grown together in ZMB1 for 20 h. After 5 min of incubation in a 1 mL microfuge tube, 100 μL of the solution was transferred to a glass slide and coverslipped. Images were obtained on a Zeiss Axio microscope and the images were analyzed with FIJI (Formerly Image J) software (NIH) with the relative fluorescent intensity obtained for each channel.

## Results

### *Clostridioides difficile* autofluorescence is influenced by various compounds

**Figure 1:**
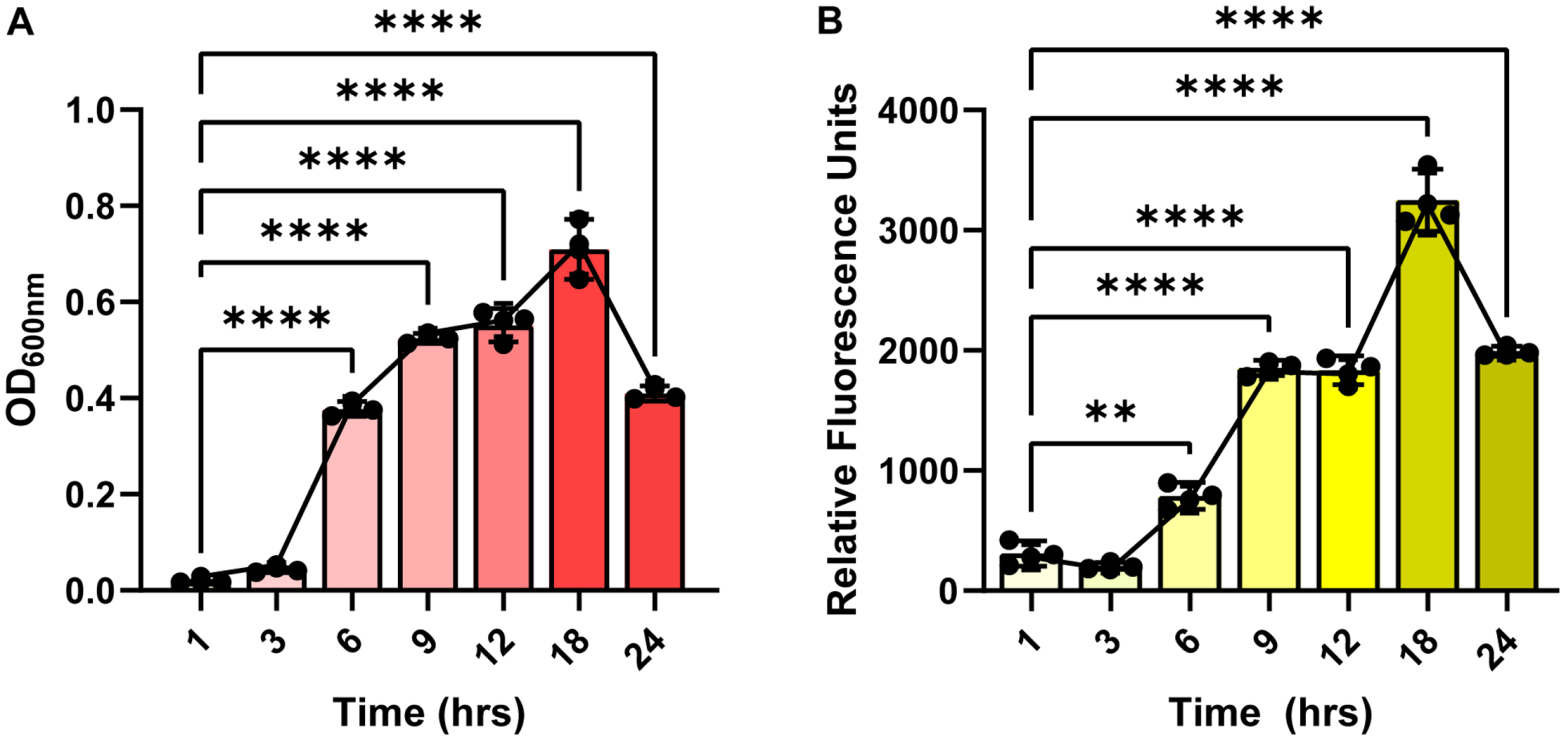
*Clostridioides difficile* autofluorescence is growth-phase dependent. *C. difficile* R20291 was grown in BHI at a starting OD_600nm_ of 0.1 and growth and fluorescence was monitored after 1, 3, 6, 9, 12, 18 and 24 h post-inoculation. **(A)** Growth was quantified by measuring OD_600_ of the culture at various time points. **(B)** Autofluorescence was quantified by measuring green fluorescence (485 nm/528 nm) of washed *C. difficile* resuspended in PBS at various time points. Data are represented as mean ± stdev. Repeated Measures ANOVA, **p < 0.01, ***p < 0.001, ****p < 0.0001.

Since the autofluorescence in *C. difficile* 630 has been shown to be growth-dependent (Oliveira Paiva et al., 2022), we first sought to determine if the autofluorescence of *C. difficile* R20291 was also growth-phase dependent. *C. difficile* R20291 was grown in BHI at a starting OD_600nm_ = 0.1 and samples were collected during lag phase (1 and 3 h), exponential phase (6 and 9 h), stationary phase (12 and 18 h), and death phase (24 h) (Figure 1). As expected, we found that *C. difficile* grew over time, with the highest growth observed after 18 h of incubation (Figure 1A). Mirroring the growth, we observed that *C. difficile* autofluorescence increased overtime, with the highest amount of autofluorescence occurring at 18 h (Figure 1B).

**Figure 2:**
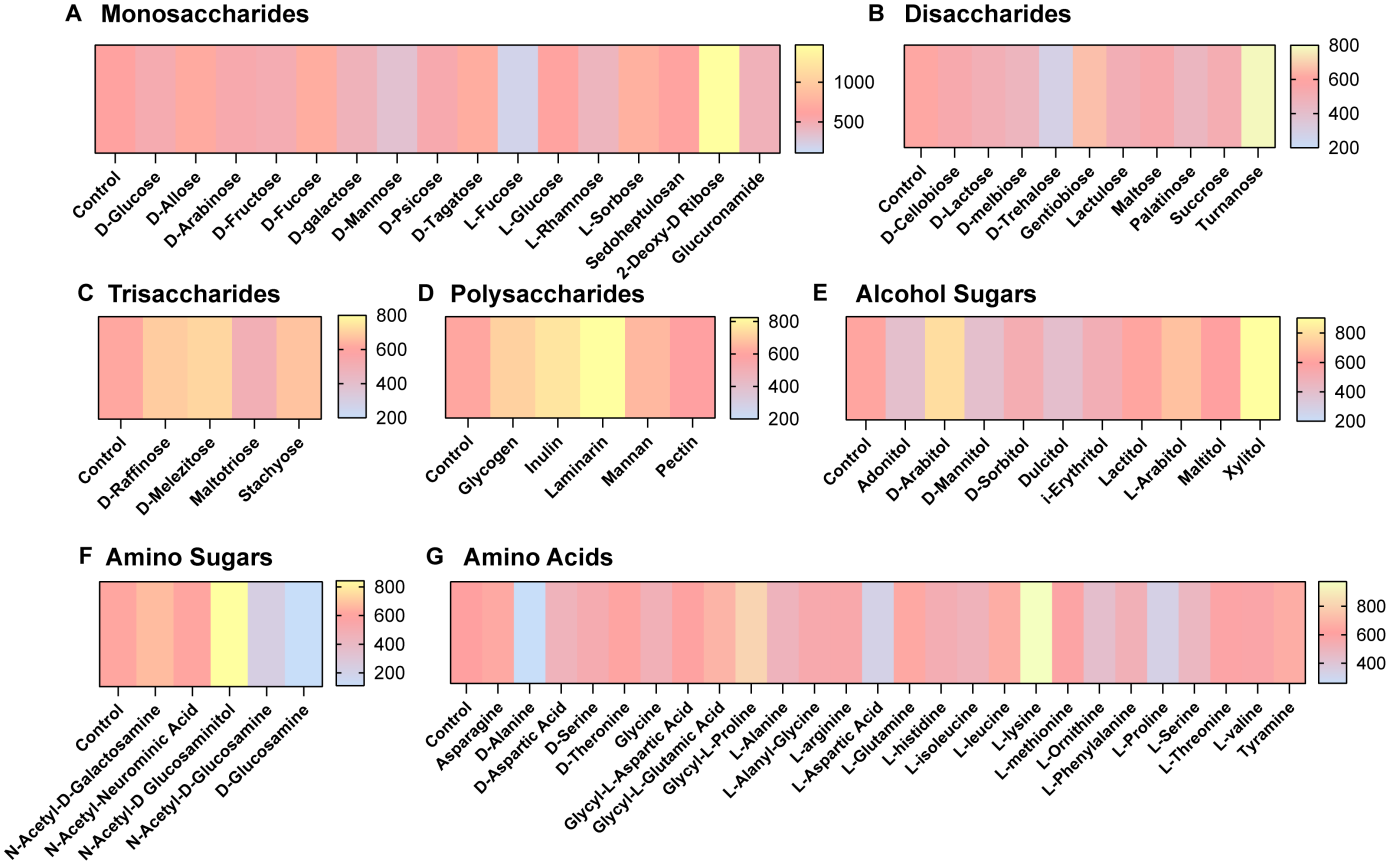
*Clostridioides difficile* autofluorescence is affected by certain sugars and amino acids. *C. difficile* was grown for 6 h in BHI anaerobically, washed with PBS and then added to Biolog^©^ Phenotypic Microarray plates in PBS at an OD_600nm_ = 1. Baseline line fluorescence and fluorescence with *C. difficile* was measured in the green channel (485 nm/528 nm). Compounds are grouped into **(A)** monosaccharides, **(B)** disaccharides, **(C)** trisaccharides, **(D)** polysaccharides, **(E)** alcohol sugars, **(F)** amino sugars, and **(G)** amino acids.

**Figure 3:**
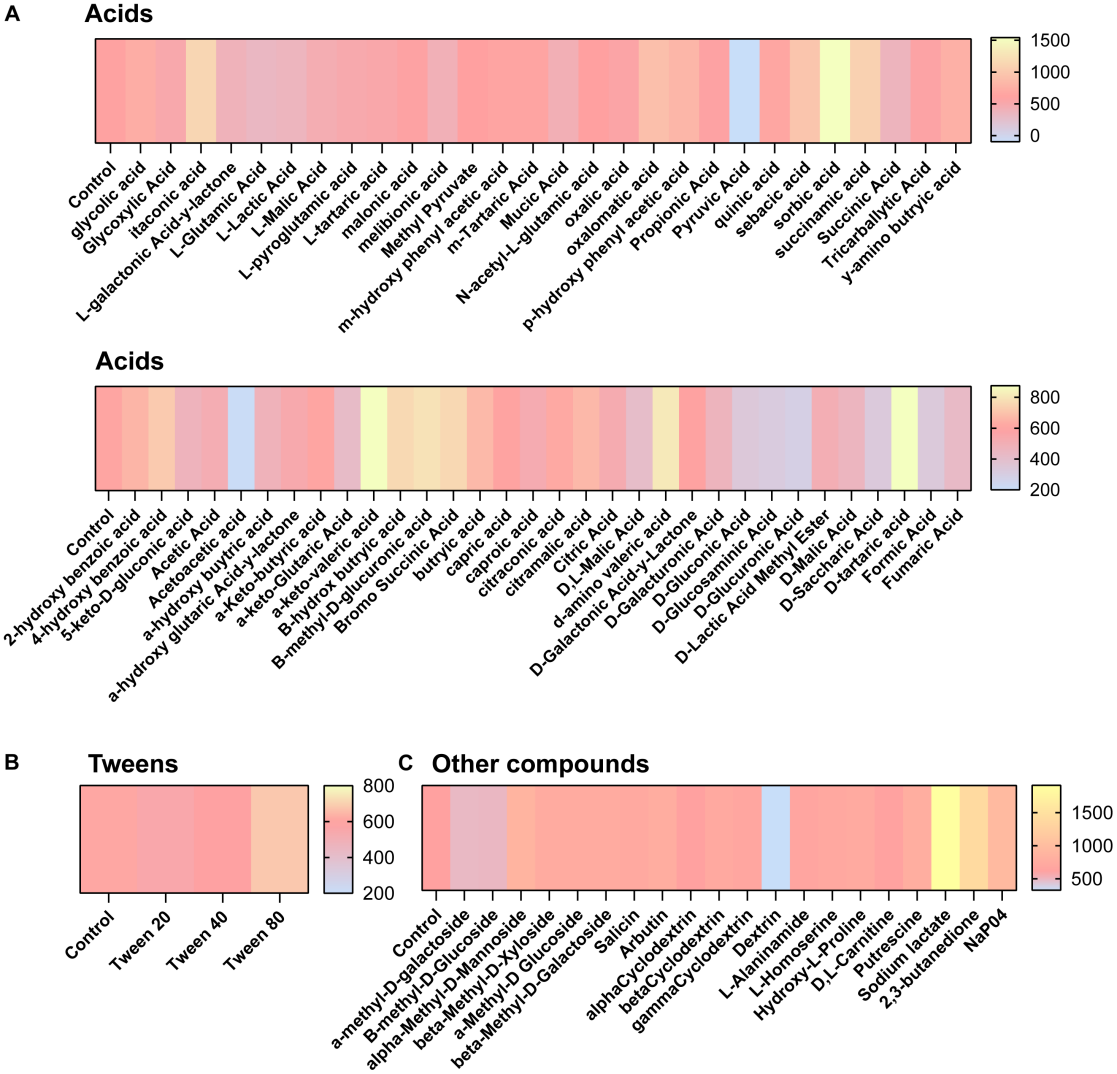
*Clostridioides difficile* autofluorescence is affected by various acids and other compounds. *C. difficile* was grown for 6 h in BHI anaerobically, washed with PBS and then added to Biolog^©^ Phenotypic Microarray plates in PBS at an OD_600nm_ = 1. Baseline line fluorescence and fluorescence with *C. difficile* was measured in the green channel (485 nm/528 nm). Compounds are grouped into **(A)** acids, **(B)** tweens, and **(C)** other compounds.

We next aimed to determine what compounds could potentially impact *C. difficile* autofluorescence. We utilized Biolog^©^ Phenotypic Microarray plates to screen a large number of compounds efficiently (Figures 2A–G; Figures 3A–C). We collected baseline levels of autofluorescence of the compounds and then added a 6 h culture of *C. difficile* R29201 in PBS at a final OD_600nm_ of 1 to the plates and monitored the fluorescence after a 5 min incubation. We observed that several compounds could increase *C. difficile* autofluorescence (Figures 2A,C–G; Figures 3A,C). The compounds that induced the highest autofluorescence in *C. difficile* were the monosaccharide 2-deoxy-ribose (2.4-fold increase) (Figure 2A), acid sorbic acid (2.5-fold increase) (Figure 3A), sodium lactate (3.1-fold increase) (Figure 3C), 2,3-butanedione (2.3-fold increase) (Figure 3C). We also found a ~ 1.5-fold increase in *C. difficile* autofluorescence in response to the disaccharide turnanose (Figure 2B), polysaccharide laminarin (Figure 2D), alcohol sugar xylitol (Figure 2E), amino sugar N-acetyl-D-Glucosaminitol (Figure 2F), amino acid L-Lysine (Figure 2G), acids α-keto-glutaric acid, D-amino valeric acid, and tartaric acid (Figure 3A). Interestingly, we also found some compounds that decreased *C. difficile* fluorescence. We found that *C. difficile* autofluorescence was lower with aspartic acid (1.8-fold decrease) (Figure 3A), dextrin (1.9-fold decrease) (Figure 3C), D-trehalose (2.2-fold decrease) (Figure 2B), D-alanine (2.4-fold decrease) (Figure 2G), N-Acetyl-D-glucosamine (2.6-fold decrease) (Figure 2F), acetoacetic acid (2.9-fold decrease) (Figure 3A), L-fucose (3.2-fold decrease) (Figure 2A), D-glucosamine (5.6-fold decrease) (Figure 2F) and pyruvic acid (6.2-fold decrease) (Figure 3A). We did not observe a pattern in the type of compounds which influenced *C. difficile* autofluorescence; we observed compounds that could increase or decrease autofluorescence in the same classes of compounds. This indicates that *C. difficile* autofluorescence is influenced by multiple types of compounds.

**Figure 4:**
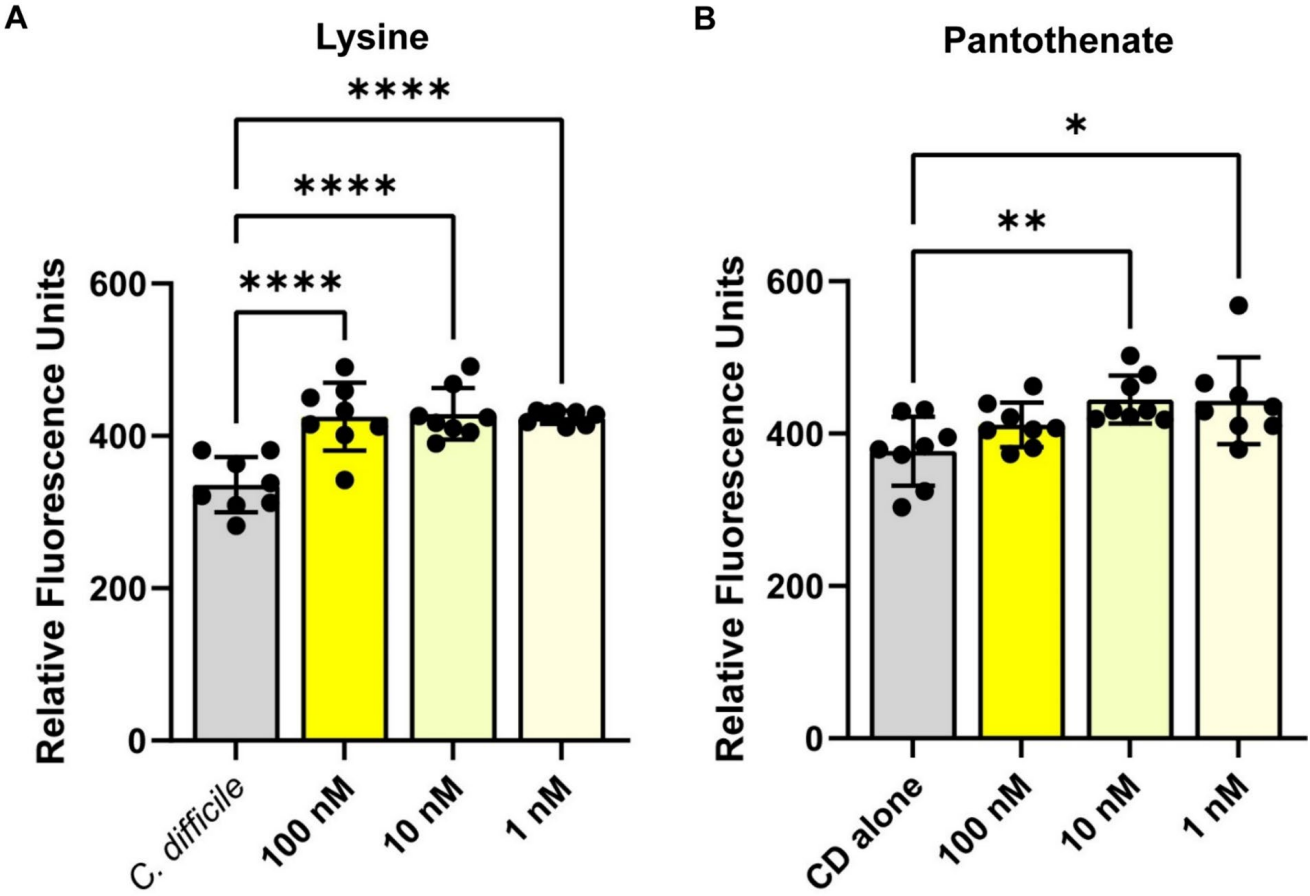
*Clostridioides difficile* autofluorescence increases in the presence of L-lysine and pantothenate. *C. difficile* was grown for 6 h in BHI anaerobically, washed with PBS and then added to various concentrations of **(A)** L-lysine or **(B)** pantothenate. Fluorescence was measured in the green channel (485 nm/528 nm). Data are represented as mean ± stdev. One Way ANOVA; *p < 0.05, **p < 0.01, ***p < 0.001, ****p < 0.0001.

To confirm the findings from the Biolog^©^ plates, we measured *C. difficile* autofluorescence with a range of concentrations from two of the compounds that showed an increase in *C. difficile* autofluorescence: L-lysine and pantothenate. Consistent with findings using the Biolog© microarrays, we saw a significant increase in *C. difficile* autofluorescence with L-lysine (Figure 4A) and pantothenate (Figure 4B) at concentrations ranging from 1 nM to 100 nM. We confirmed *C. difficile*’s autofluorescence by confocal microscopy (Supplementary Figure S2A). When we quantified the fluorescence of individual bacteria in confocal images, we observed cell to cell variation in the level of autofluorescence, with an overall increase in autofluorescence in the bacteria treated with 1 mM L-lysine and 1 mM pantothenate (Supplementary Figures S2B,C). These data support the finding that specific compounds can enhance *C. difficile* autofluorescence.

### Gut bacteria enhance *Clostridioides difficile* autofluorescence

**Figure 5:**
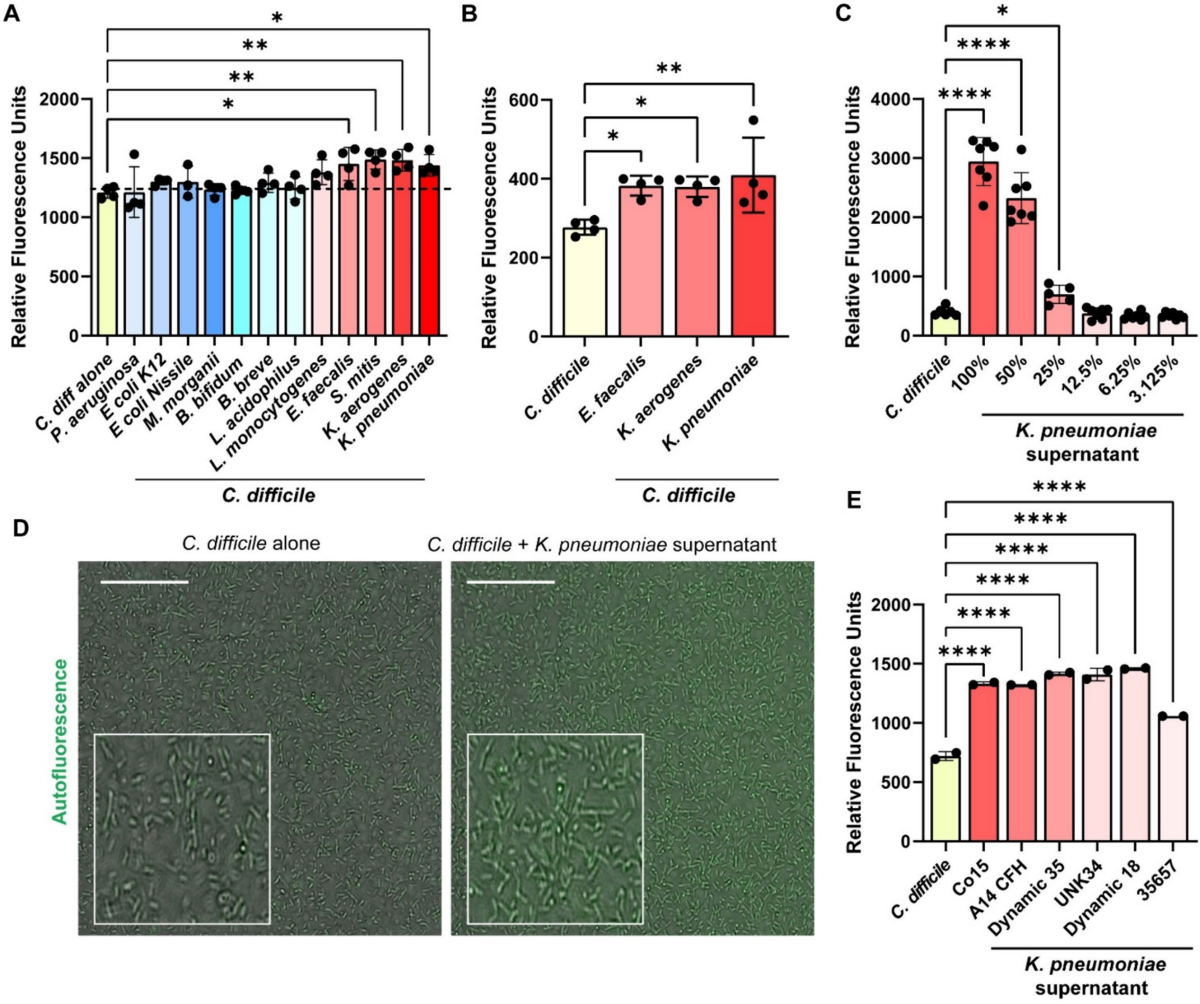
Bacterial metabolites increase *C. difficile autofluorescence.* **(A)** *C. difficile* was grown in a fully defined medium, ZMB1, supplemented with 10% of cell-free conditioned media from other bacteria. Green autofluorescence was measured in washed *C. difficile* cells after culturing for 20 h on a fluorescent plate reader (485 nm/528 nm). **(B)** *C. difficile* was grown for 6 h in BHI anaerobically, washed with PBS and then added to 25% of *E. faecalis* ATCC 35038, *K. aerogenes* ATCC 35029, and *K. pneumoniae* ATCC 35657 cell free conditioned media. Green autofluorescence was measured in washed *C. difficile* cells on a plate reader (485 nm/528 nm). **(C)** Green fluorescence was measured by a plate reader after *C. difficile* was incubated with various concentrations of *K. pneumoniae* ATCC 35657 conditioned media. **(D)** Representative fluorescent images of *C. difficile* alone or *C. difficile* after incubation with 50% *K. pneumoniae* ATCC 35657 cell free supernatant. Scale bar = 50 μm. **(E)** Green autofluorescence was measured after *C. difficile* by a plate reader was exposed to conditioned media from various *K. pneumoniae* strains after incubation. Data are represented as mean ± stdev. One Way ANOVA; *p < 0.05, **p < 0.01, ***p < 0.001, ****p < 0.0001.

Several of the compounds we identified in the Biolog^©^ microarrays that could induce *C. difficile* autofluorescence can be synthesized by bacteria. To investigate if bacterial metabolites could influence *C. difficile* autofluorescence, we grew *C. difficile* in cell-free bacterial conditioned media from various bacteria that can be found in the human gut (Figure 5). We found that that the metabolites from commensal *E. coli, M. morganii, B. bifidum, B. breve* and *L. acidophilus* did not affect *C. difficile* autofluorescence (Figure 5A). We also found that the classically pathogenic *P. aeruginosa* and *L. monocytogenes* metabolites did not influence autofluorescence in *C. difficile*. However, we did observe that metabolites from *E. faecalis*, *S. mitis*, *K. aerogenes*, and *K. pneumoniae* significantly increased overall *C. difficile* autofluorescence (Figure 5A). This effect was independent of *C. difficile* growth or culture pH, since *C. difficile* grew to a similar degree with all the bacterial supernatants and the final pH of *C. difficile*-supernatant culture was unchanged (Supplementary Figures S1A,B). To identify if *C. difficile* autofluorescence was increased rapidly by bacterial metabolites, we generated cell-free conditioned medium from *E. faecalis, K. aerogenes* and *K. pneumoniae* and examined *C. difficile* autofluorescence to these secreted metabolites. We found that all three of these cell-free supernatants could elevate *C. difficile* autofluorescence (Figure 5B). These results suggest that bacteria produce compounds that can increase *C. difficile* autofluorescence.

### *Klebsiella pneumoniae* isolates enhance *Clostridioides difficile* autofluorescence

*K. pneumoniae* is an opportunistic bacterial species that is normally found in low abundance in the healthy human gut, but can increase substantially with antibiotics or in the setting of inflammation as one signature of a perturbed microbiota (Lepuschitz et al., 2020; Hudson Andrew et al., 2022). Studies have shown that *K. pneumoniae* relative abundance is increased in the feces of patients with *C. difficile* infection (Giuliano et al., 2014; Bruno et al., 2018; Hong et al., 2019; Golubovska et al., 2021). As a result, we decided to take a closer look at *C. difficile* autofluorescence with *K. pneumoniae*. We observed that *C. difficile* autofluorescence increased in a dose-dependent manner to the concentration of *K. pneumoniae* ATCC 35657 cell-free supernatant (Figure 5C). We also confirmed the increase in *C. difficile’s* autofluorescence in response to 50% of *K. pneumoniae* ATCC 35657 supernatant by microscopy (Figure 5D). When we quantified the fluorescence of individual *C. difficile* cells to *K. pneumoniae* metabolites by confocal microscopy, we found an overall increase in autofluorescence; although we observed a range of fluorescence in *C. difficile* bacteria (Supplementary Figures S3A,B). To determine if other *K. pneumoniae* strains could impact *C. difficile* autofluorescence, we obtained intestinal isolates of *K. pneumoniae* (C015, A14 CFH, A05E, Dynamic 35, UNK34, and Dynamic 18) and included another commercially available *K. pneumoniae* strain (CB1) in our assays. We generated cell-free conditioned media from these *K. pneumoniae* strains and found that *C. difficile* increased its overall autofluorescence when exposed to these *K. pneumoniae* supernatants for 5 min (Figure 5E). These results suggest that *K. pneumoniae* metabolites increase *C. difficile* autofluorescence, and that this effect is conserved across *K. pneumoniae* strains.

**Figure 6:**
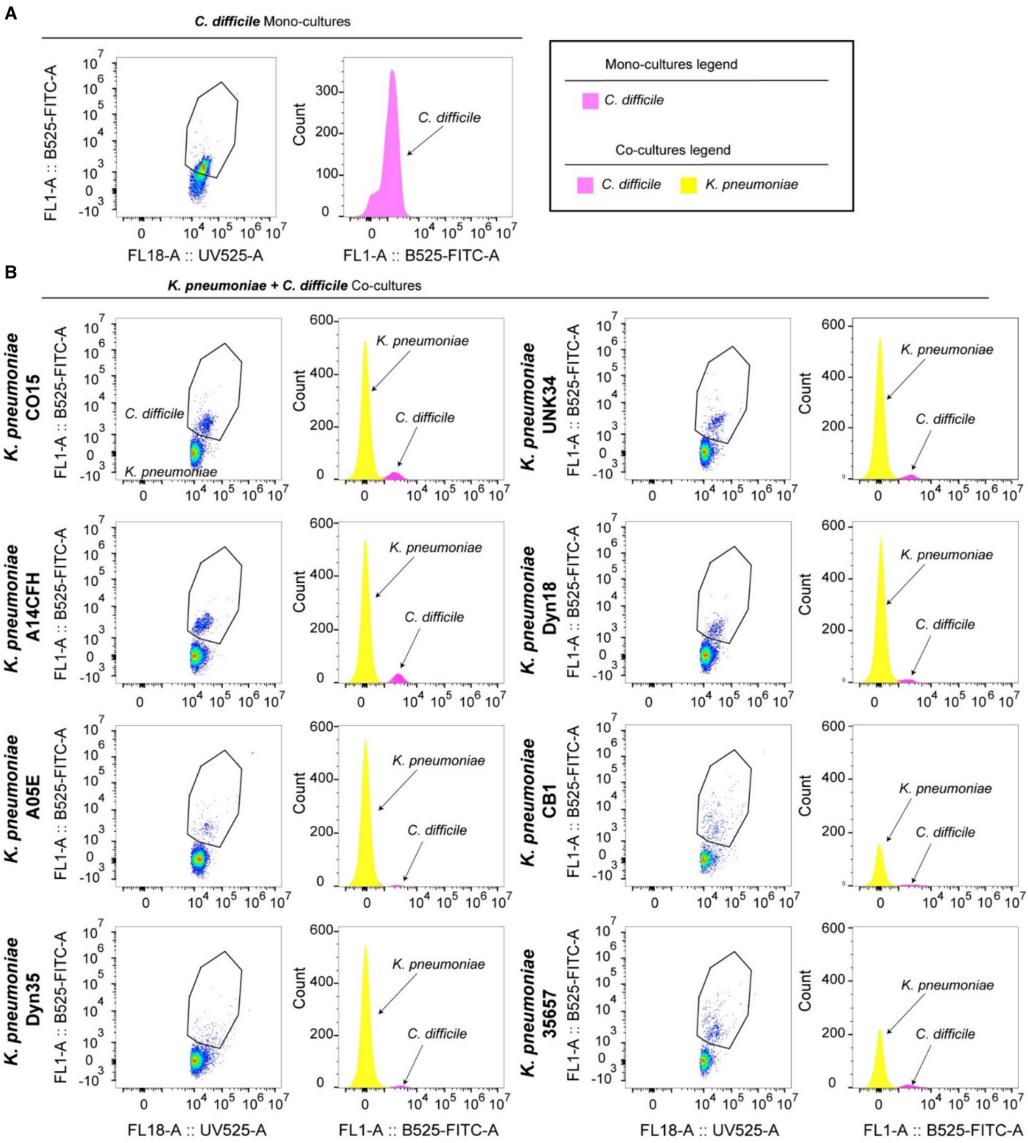
*Clostridioides difficile* autofluorescence can distinguish *C. difficile* in co-culture with *K. pneumoniae. C. difficile* and *K. pneumoniae* strains were grown as a co-cultures in ZMB1 for 20 h, stained with Hoechst and examined by flow cytometry. The data depicts a representative gating strategy for identifying highly autofluorescent *C. difficile* in the green channel and histograms of the quantified fluorescence. **(A)** Flow analysis of *C. difficile* monocultures. **(B)** Flow cytometry analysis of *C. difficile* and *K. pneumoniae* co-cultures.

We next sought to determine if *K. pneumoniae* could increase *C. difficile* autofluorescence when the bacteria are grown together. We grew *C. difficile* and *K. pneumoniae* in mono- and co-cultures in ZMB1 for 20 h. We found that *C. difficile* was present in all the co-cultures by qPCR (Supplementary Figure S4A). We also confirmed that *C. difficile* was autofluorescent in the co-cultures of *C. difficile* and *K. pneumoniae* ATCC 35657 by microscopy (Supplementary Figure S4B). We then performed flow cytometry on mono-cultures and co-cultures of *C. difficile* and *K. pneumoniae*. We found that all the *K. pneumoniae* strains had undetectable levels of autofluorescence (Supplementary Figure S5). When we grew *C. difficile* and *K. pneumoniae* together for 20 h, we could detect two populations of cells. Since *K. pneumoniae* does not exhibit green autofluorescence, any cells that were positive for green fluorescence were identified to be *C. difficile*. We observed that *C. difficile* had high green fluorescence when grown in co-culture with all strains of *K. pneumoniae* tested and this fluorescence could distinguish *C. difficile* from *K. pneumoniae* (Figure 6). Collectively these findings suggest that *C. difficile’s* autofluorescence is influenced by dietary compounds and bacterial metabolites and that the increase in *C. difficile* autofluorescence could potentially allow researchers to identify *C. difficile* in mixed bacterial populations.

## Discussion

*C. difficile* displays intrinsic green autofluorescence, but the factors that influence this autofluorescence have not been fully elucidated. In this study, we sought to identify compounds and conditions that could enhance *C. difficile* autofluorescence. We found that several compounds elevated *C. difficile* autofluorescence, including 2-deoxy-D-Ribose, turnanose, laminarin, L-lysine, N-acetyl-D-glucosaminitol, xylitol, sorbic acid, α-keto-valeric acid, D-amino valeric acid, D-tartaric acid, sodium lactate, 2,3-butanedione and pantothenate. We also found that bacteria can produce compounds which increase autofluorescence in *C. difficile*. Interestingly, we observed that *K. pneumoniae* strains were highly efficient at upregulating autofluorescence; so much so that we could grow *C. difficile* with *K. pneumoniae* and measure autofluorescence by flow cytometry. These findings expand our knowledge of *C. difficile* autofluorescence.

*C. difficile* autofluorescence has been reported by several groups. Ransom et al. first reported *C. difficile*’s green autofluorescence in 2015 while studying a method to fluorescently label *C. difficile* with minimal oxygen required to mature the chromophore (Ransom Eric et al., 2015). Since then, other groups have utilized this autofluorescence to visualize *C. difficile* in various capacities. Garcia-Garcia et al. paired *C. difficile* autofluorescence images with SNAP-PrkC staining (Garcia-Garcia et al., 2022). The widespread autofluorescence allowed for the localization of SNAP-PrkC to be visualized at both stationary growth phase, as well as sporulation phase of *C. difficile*. Kint et al. also used this autofluorescence as a tool to image *C. difficile* to then show localization of other targets (Kint et al., 2020). In this study, we expand the utility of using *C. difficile* autofluorescence to flow cytometry. We found that when *C. difficile* was grown with *K. pneumoniae* strains, it became highly autofluorescence and we could distinguish *C. difficile* and *K. pneumoniae* by flow cytometry. This method provides an alternative validation of *C. difficile* growth with other bacteria and could prove valuable for other studies involving *C. difficile*-microbe cross-talk. Previous studies have observed the beneficial relationship between *C. difficile* and other bacteria, such as Enterococci (Smith et al., 2022) and Fusobacteria (Engevik et al., 2021) so this technique offers another method to observe these relationships.

Consistent with other groups, we found that *C. difficile* autofluorescence increased with growth (Ransom Eric et al., 2015). Growth phase-dependent autofluorescence has also been observed in other bacteria, including *Escherichia coli* or *Bacillus pumilus* (Manzo et al., 2013; Mihalcescu et al., 2015). We opted to examine autofluorescence in *C. difficile* after 6 h of growth because this point had a lower baseline level of autofluorescence. This allowed us to monitor increases in autofluorescence in response to various factors. To the best of our knowledge, no group to date has shown that different bacteria and their metabolites can influence *C. difficile*’s autofluorescence. In this study we demonstrated that compounds potentially produced by bacteria and bacterial secreted metabolites can influence *C. difficile* autofluorescence. One of the interesting compounds we identified that elevated *C. difficile* autofluorescence of L-lysine. We found that several concentrations of L-lysine increased *C. difficile’s* autofluorescence. Certain bacteria are able to produce L-lysine, including *Corynebacterium glutamicum* (Nærdal et al., 2017), *Bacillus methanolicus* (Nærdal et al., 2017), various rumen bacteria (Styriak et al., 1992), and *K. pneumoniae* (Gilani et al., 2023). We speculate that L-lysine produced by *K. pneumoniae* could be responsible for the elevated levels of *C. difficile* autofluorescence that we observed with *K. pneumoniae* supernatant and co-cultures.

In addition to L-lysine, we also observed that pantothenate could increase *C. difficile’s* fluorescence. Pantothenate has been found in other bacteria to elevate glutathione and promote antibiotic resistance (Yan et al., 2023). It is not clear how pantothenate enhances autofluorescence, but it could be due to alterations in cell stress. A link between autofluorescence and oxidative stress has been proposed in *Bacillus* (Manzo et al., 2013) and *E. coli* (Mihalcescu et al., 2015). Others have found that autofluorescence can be enhanced in bacteria under antibiotic stress (Renggli et al., 2013) and has been correlated with an increase in expression of flavin biosynthesis pathways (Surre et al., 2018). In future, it would be interesting to examine *C. difficile* stress upon exposure to the autofluorescence inducing compounds identified in our study.

In this study we observed an overall increase in fluorescence intensity. This was observed by assessing the population on a fluorescent plate reader and by quantifying the fluorescence of individual bacteria by confocal microscopy. Interestingly we did identify variation among individual *C. difficile* cells in response to L-lysine, pantothenate and *K. pneumoniae* metabolites. Populations of genetically identical cells growing under uniform conditions can exhibit cell-to-cell heterogeneity in gene expression. Oliveira Paiva et al., 2022, used a similar single-cell autofluorescence analysis to examine *C. difficile* fluorescence and found that *C. difficile* had an increase in the average fluorescence intensity during the stationary growth phase, but there was significant heterogeneity between cells (Oliveira Paiva et al., 2022). Our data suggests an overall increase in autofluorescence intensity across all cells, although some cells did respond more than others. It is possible that a sub-population of *C. difficile* cells were better poised to respond to certain compounds and these cells exhibited a more rapid increase in autofluorescence. In the future it would be interesting to examine individual bacterial cells to further identify potential sub-populations of *C. difficile*.

A limitation of this study was that we were limited in the bacteria we could grow with *C. difficile* in co-culture to observe the effect on *C. difficile* autofluorescence. Bacteria such as *P. aeruginosa* (Zhang et al., 2024) and *E. coli* (Renggli et al., 2013) display their own autofluorescence. As such, we avoided using these bacteria to focus solely on *C. difficile*. We were then further limited by bacteria that would allow both itself and *C. difficile* to grow in co-culture. In a pilot experiment, we observed that several commensal bacteria out-competed *C. difficile*. As a result, we chose to focus on *K. pneumoniae* since the co-cultures supported the growth of both *C. difficile* and *K. pneumonia*. Future studies could potentially look at other bacterial co-culture pairs to see if other species enhance or diminish *C. difficile* autofluorescence.

In conclusion, our data sheds light on different factors that influence *C. difficile* autofluorescence, such as growth time in culture and compounds found in the human diet or produced by other gut bacteria. We specifically identified that L-lysine and *K. pneumoniae* could upregulate *C. difficile* autofluorescence. More in-depth studies will be needed to further understand the pathways being affected that lead to *C. difficile* autofluorescence, however these findings highlight a facet of the complexity of bacterial interactions.

## Data Availability

The raw data supporting the conclusions of this article will be made available by the authors, without undue reservation.
