# Supplementary material for: Bacterial metabolites influence the autofluorescence of Clostridioides difficile
Source: Front Microbiol. 2024 Oct 8;15:1459795. doi: 10.3389/fmicb.2024.1459795 (PMC11493716; doi:10.3389/fmicb.2024.1459795)
Supplement: Supplementary file 1 [file Data_Sheet_1.pdf]

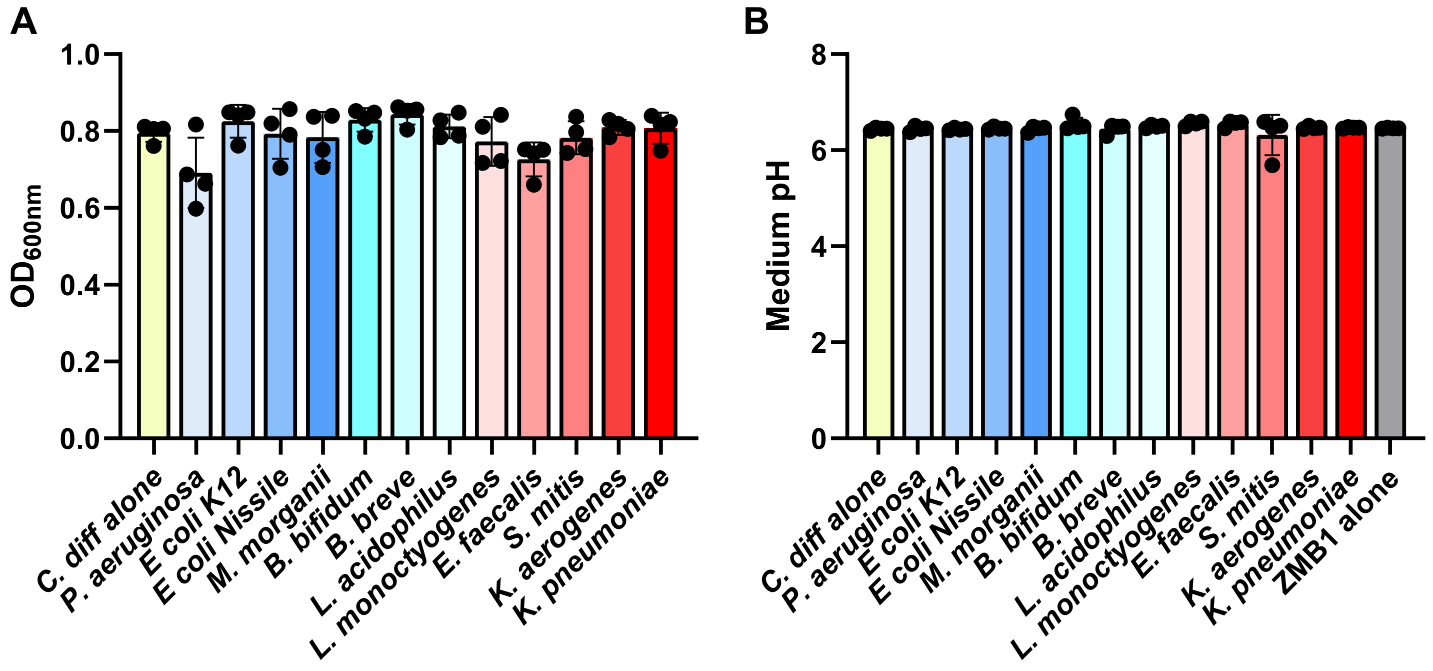
A
B
OD600nm
Medium pH
0.0
0.2
0.4
0.6
0.8
1.0
0
2
4
6
8
C. diff alone
P. aeruginosa
E coli K12
E coli Nissile
M. morganii
B. bifidum
B. breve
L. acidophilus
L. monocytogenes
E. faecalis
S. mitis
K. aerogenes
K. pneumoniae
ZMB1 alone

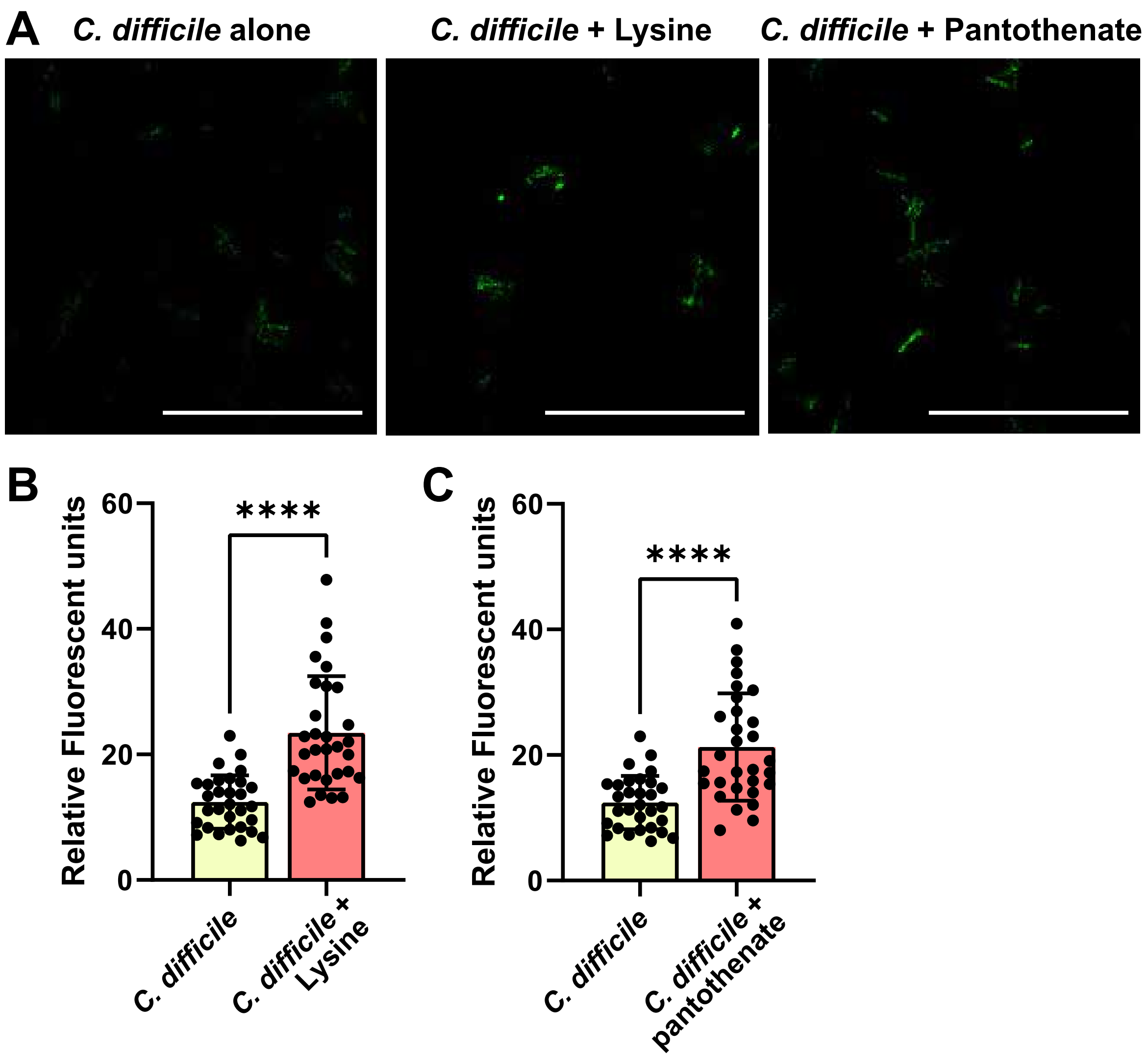

A
C. difficile alone
C. difficile + Lysine
C. difficile + Pantothenate
B
Relative Fluorescent units
60
40
20
0
****
C. difficile
C. difficile + Lysine
C
Relative Fluorescent units
60
40
20
0
****
C. difficile
C. difficile + pantothenate

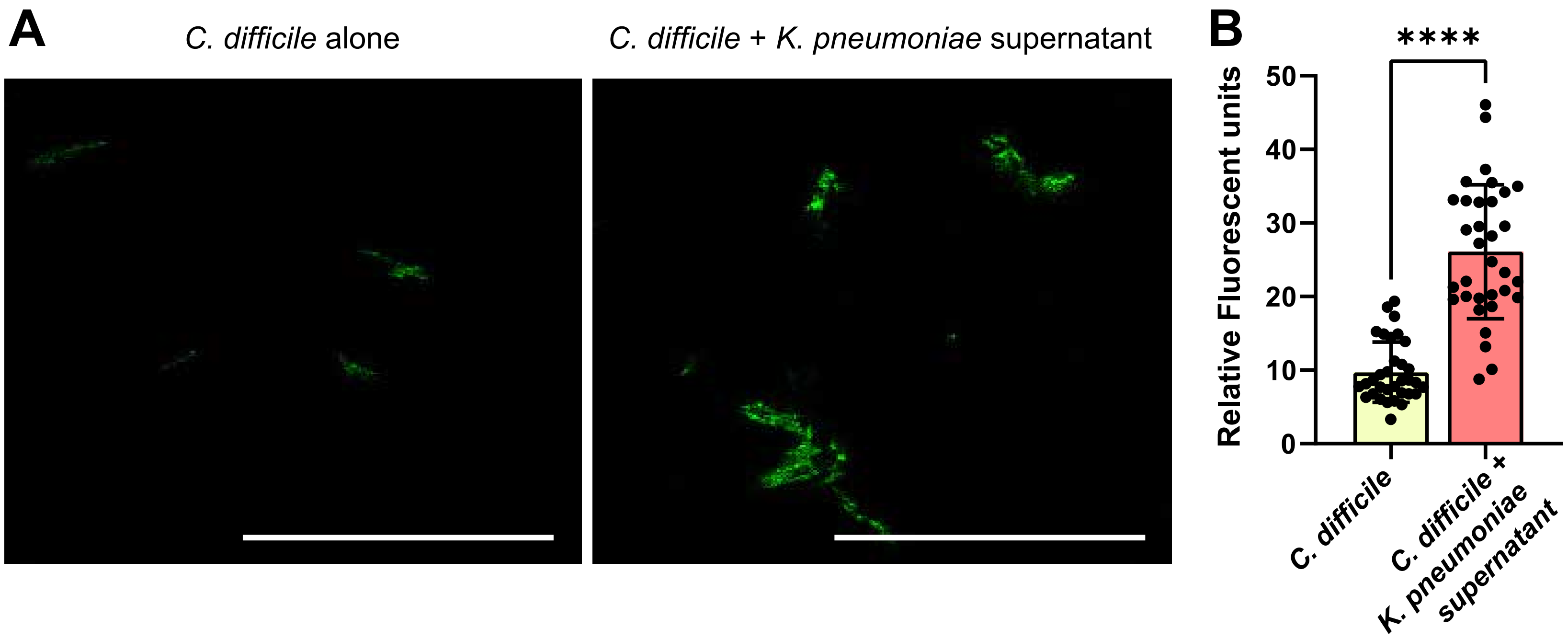

A
C. difficile alone
C. difficile + K. pneumoniae supernatant
B
****
Relative Fluorescent units
50
40
30
20
10
0
C. difficile
C. difficile + K. pneumoniae supernatant

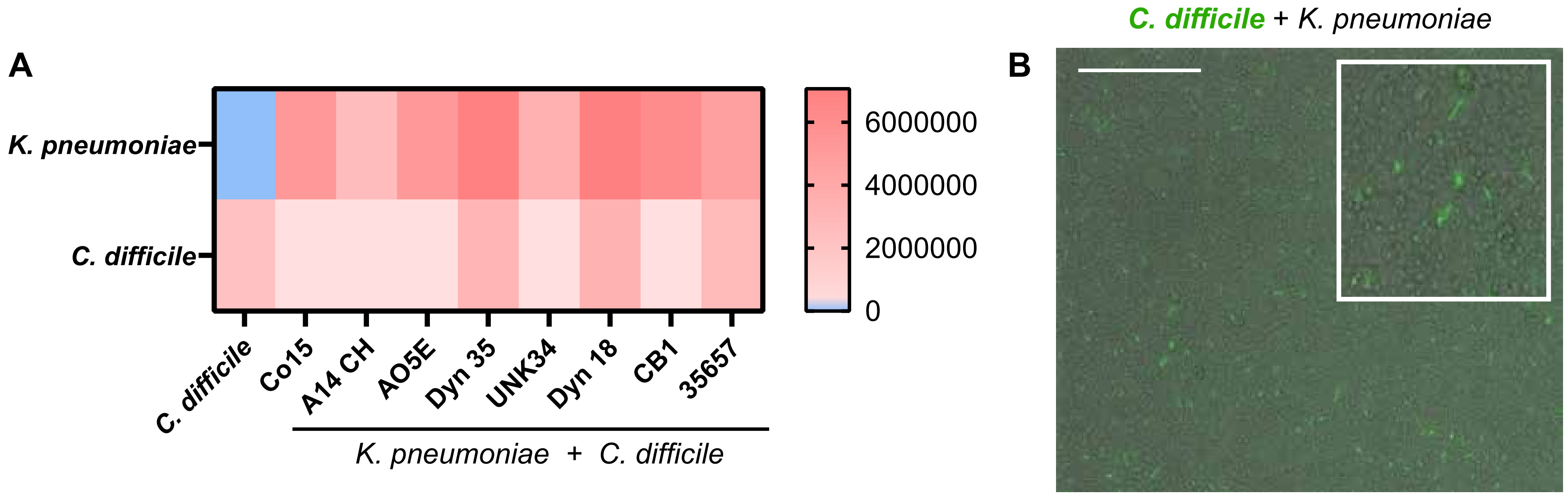
A
K. pneumoniae
C. difficile
6000000
4000000
2000000
0
C. difficile
Co15
A14 CH
AO5E
Dyn 35
UNK34
Dyn 18
CB1
35657
K. pneumoniae + C. difficile
B
C. difficile + K. pneumoniae

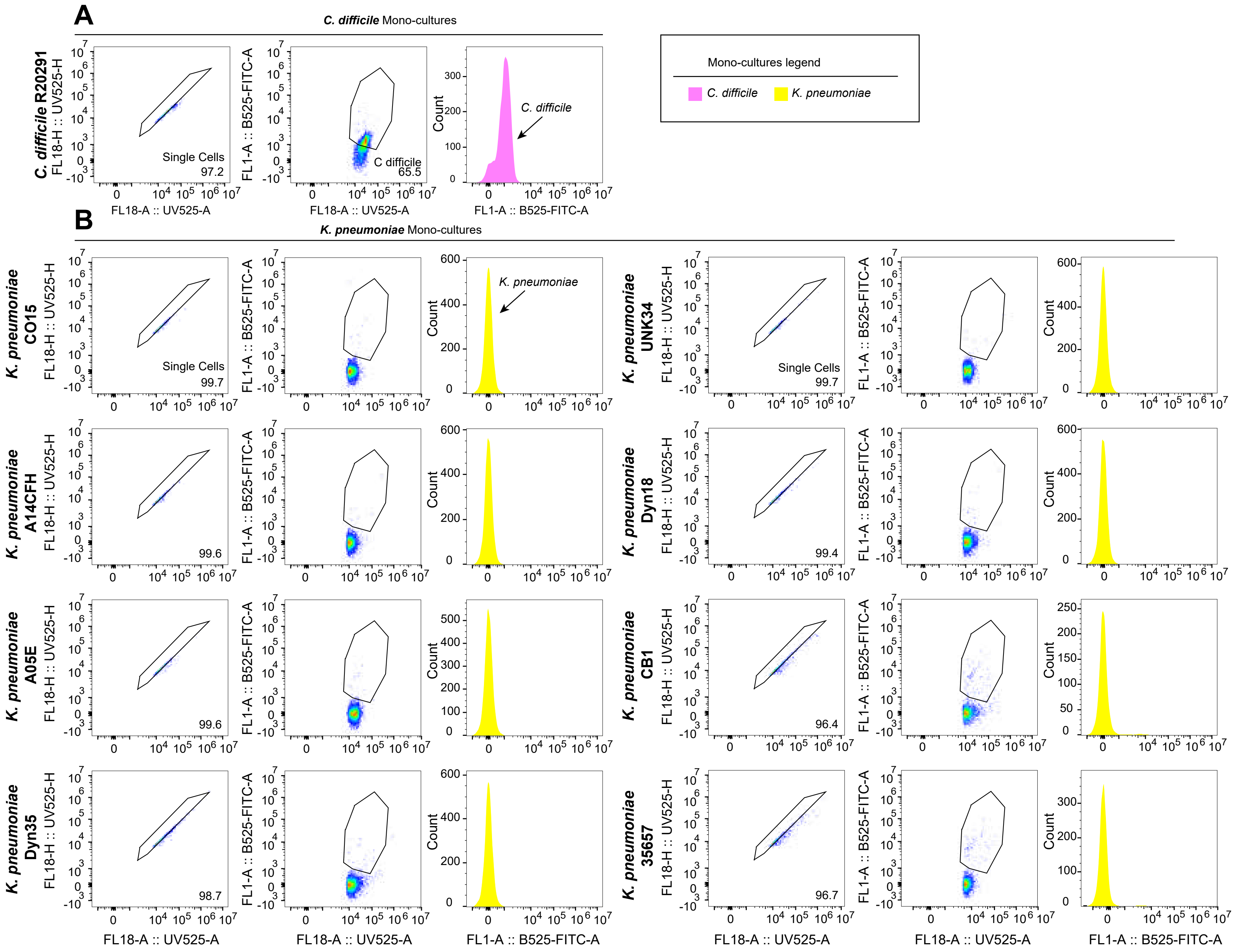
A
C. difficile Mono-cultures
C. difficile R20291
Single Cells
97.2
C difficile
65.5
C. difficile
Mono-cultures legend
C. difficile
K. pneumoniae
B
K. pneumoniae Mono-cultures
K. pneumoniae CO15
K. pneumoniae UNK34
K. pneumoniae A14CFH
K. pneumoniae Dyn18
K. pneumoniae A05E
K. pneumoniae CB1
K. pneumoniae Dyn35
K. pneumoniae 35657
FL18-H :: UV525-H
FL18-A :: UV525-A
FL1-A :: B525-FITC-A
Count
99.7
99.6
99.4
96.4
98.7
96.7

Supplemental Figure Legends

Supplemental Figure 1*: C. difficile growth and pH is not affected by bacterial conditioned media. C. diffiicle* was grown for 20 hrs in ZMB1 with 10% cell-free conditioned media from various gut bacteria. (**A**) *C. difficile* growth was quantified by measuring OD_{600} of the cultures at the 20 hour time point. (**B**) Bacterial culture pH was quantified via litmus assay at the 20 hour time point. Data are represented as mean ± stdev.

Supplemental Figure 2. *Confocal images of C. difficile reveal variable but elevated autofluorescence in response to L-lysine and pantothenate.* (**A**) *C. difficile* was grown for 6 hrs and then incubated with mM L-lysine or 1 mM pantothenate. Representative images of *C. difficile* autofluorescence by confocal microscopy at 63x. Scale bar = 50 µm. (**B,C**) Quantification of autofluorescence at the level of individual bacteria incubated with (**B**) L-lysine and (**C**) pantothenate. Data are represented as mean ± stdev. Student t-test; ****p<0.0001.

Supplemental Figure 3. *Confocal images of C. difficile reveal variable but elevated autofluorescence in response to K. pneumoniae cell-free supernatant.* (**A**) *C. difficile* was grown for 6 hrs and then incubated with *K. pneumoniae* cell-free supernatant. Representative images of *C. difficile* autofluorescence by confocal microscopy at 63x. Scale bar = 50 µm. (**B**) Quantification of autofluorescence at the level of individual bacteria. Data are represented as mean ± stdev. Student t-test; ****p<0.0001.

Supplemental Figure 4*: C. difficile and K. pneumoniae grow together in co-culture. C. difficile* and *K. pneumoniae* strains were grown together in ZMB1 for 20 hours. (**A**) qPCR was performed to quantify the bacterial levels in culture and calculated to CFU based on a standard curve. (**B**) Representative images of *C. difficile* and *K. pneumoniae* ATCC 35657 co-cultures depicting autofluorescent *C. difficile* (green) with non-autofluorescent *K. pneumoniae* with DIC. Scale bar = 50 µm.

Supplemental Figure 5*: K. pneumoniae strains are not autofluorescent. K. pneumoniae* strains were grown as a monoculture in ZMB1 for 20 hours, stained with Hoechst and examined by flow cytometry. The data depicts a representative gating strategy for identifying highly autofluorescent bacteria in the green channel and histograms of the quantified fluorescence.
